# Bleb-related Endophthalmitis: Clinical Presentation, Isolates, Treatment and Visual Outcome of Culture-proven Cases

**DOI:** 10.4103/0974-9233.48862

**Published:** 2009

**Authors:** Basel T. Ba'arah, William E. Smiddy

**Affiliations:** Department of Ophthalmology, University of Miami, Bascom Palmer Eye Institute, Miami, Florida, United States of America

**Keywords:** Bleb-related Endophthalmitis, Antibiotics, Vitrectomy

## Abstract

**Purpose:**

To investigate clinical features, causative organisms and their antibiotic sensitivity, management, and visual acuity outcomes of eyes with bleb-related endophthalmitis (BRE).

**Design:**

Retrospective, noncomparative, consecutive eye series.

**Methods:**

Clinical and microbiological records of patients with culture positive bleb-related endophthalmitis treated at a single institution between April 1995 and February 2002 were revised retrospectively.

**Main Outcome Measures:**

Final visual acuity, loss of eye and complications.

**Results:**

There were 34 cases with presenting visual acuities ranging from 20/200 to light perception. Decrease of visual acuity was the most frequent sign (94%) followed by pain (79%) and hypopyon (53%). Associated features included pseudophakia (79%), vitreous wick (29%), and wound leak (12%). The most frequent organisms isolated from vitreous specimens, were streptococcus species (55%) and gram positive coagulase negative staphylococci (20%). Polymicrobial growth was noted in 27% of cases. The cultured organisms were sensitive to antibiotics used in 94% of cases. Treatment modality used was vitreous tap with antibiotic injection without (65%) or with vitrectomy (35%). The most common intravitreal antibiotics combination was vancomycin with ceftazidime, Intravitreal dexamethsone was administered in 56% of cases. Final visual outcome of 20/400 or better was noticed in 50% of cases without and 33% with vitrectomy, but this was not statistically significant (p=0.45). The difference in final visual acuity of cases infected by gram-positive coagulase-negative staphylococci and streptococcus species were not statistically significant (p= 0.18). Overall, final visual outcome of 20/400 or better was noticed in 47% of cases, while no light perception was recorded in 8 (24%) cases. Of no light perception cases 7 underwent evisceration or enucleation. Overall, 32% of the cases experienced other complications like retinal detachment with dislocated intraocular lens, phthisis bulbi, and epiretinal membrane formation.

**Conclusion:**

BRE is associated with substantial visual morbidity. Prompt treatment of BRE with intravitreal vancomycin and broad spectrum antibiotics recommended while culture results are pending. Neither tap-injection with vitrectomy nor tap-injection without vitrectomy proved superior in the management of this condition.

Bleb-related endophthalmitis (BRE) is the second most frequent (16.7%) cause of postoperative endophthalmitis after acute and chronic post-cataract surgery endophthalmitis.^1^ It's incidence is reported to be between 0.2% to 1.3%,^2^,^3^ and is more common with the use of antiproliferative agent (up to 3%) and even higher when the bleb is placed inferiorly (up to 9.4%).^4^–^7^ Like other forms of endophthalmitis, BRE usually presents with decreased visual acuity, redness, pain, diffuse conjunctival congestion, opalescent blebs with intense fibrin and/or hypopyon in the anterior chamber, and florid vitritis.^8^,^9^ These signs may be influenced by different variables and factors such as time to initial treatment, causative organism, wound leak and the presence of a vitreous wick. The most commonly reported microorganisms cultured from the vitreous of eyes with BRE are staphylococcal species^10^,^11^ and streptococcal species.^12^,^13^ The Endophthalmitis Vitrectomy Study (EVS) compared the results of two different management approaches for post-cataract surgery endophthalmitis, but did not address BRE which has specific features that may require alterations in management.^14^

The purpose of the current study is to analyze the clinical features, causative organisms with their antibiotic sensitivity, management and visual acuity outcomes of eyes treated for BRE.

## METHODS

This study protocol was approved by the institutional review board of the University of Miami School of Medicine. Medical and microbiological records of patients with culture positive BRE treated at Bascom Palmer Eye Institute between April 1995 and February 2002 were reviewed retrospectively. Patients were included in the study if they had clinical signs of endophthalmitis, positive intraocular bacteriological culture and visual acuity of light perception or better at the initial examination.

The data recorded at clinical presentation included age, gender, best corrected visual acuity (BCVA), interval from previous surgery, timing of initial treatment, and ocular symptoms of pain, time to decrease of vision, surgical wound complication, intraocular lens style (if present), pupillary reaction, or hypopyon presence and its size.

Intraoperative data included type of procedure; vitreous tap and intravitreal injection with (tap-PPV-injection group) or without vitrectomy (tap-injection group), intravitreal, subconjunctival, systemic or topical agent used and treatment setting (outpatient vs. Inpatient).

Post-procedure data included the results of vitreous and aqueous cultures growth, sensitivity for used antibiotics, vitreous re-injection, re-operation (especially enucleation or evisceration), final BCVA, complications, and duration (if any) of inpatient stay were noted.

Cultured organisms were grouped into 5 categories including; gram-positive coagulase negative staphylococcus species, gram-positive coagulase positive staphylococcus, gram-positive streptococcus species, gram-positive bacilli, and gram-negative species.

Intravitreal agents included: vancomycin (1mg), ceftazidime (2.25mg), gentamicin (0.1mg), or decadron (0.4mg) delivered in a 0.1ml volume. Subconjunctival agents (when used) were administered in 0.5ml volume of vancomycin (50mg), ceftazidime (50mg), gentamicin (20mg), or dexamethasone (4.0mg). Systemic intravenous antibiotics (when used) included ceftazidime 1.0g Q12 hours, vancomycin 500mg Q8 hours, and oral ciprofloxacin 500mg Q 12hr. Topical antibiotics included vancomycin (50mg/ml), ceftazidime (50mg/ml), tobramycin (14mg/ml), or gentamicin (14mg/ml) and prednisolone acetate 1%.

Statistical analyses for proportional data were done with two-tailed Fisher's exact test to detect group differences.

The main outcome measures included loss of eye (enucleation or evisceration), severe loss of vision (no light perception), complications, and final BCVA ≥ 20/400.

## RESULTS

One hundred and two cases of culture positive bacterial endophthalmitis of all types were recovered from the microbiological records including pseudophakia (51%, 52 cases), bleb-related (BRE) (34%, 35cases), trauma, and other categories (6%, 9% respectively). The 35 BRE culture positive cases constituted the cohort for the current study. One case was excluded because of no light perception vision at initial examination.

Patient characteristics at presentation are summarized in Table 1. BRE developed at a mean of 51.5 ± 58 months after filtration surgery (range, 0.03 -249.3). Acute or early BRE (< 2weeks) observed in 15% of cases. Decreased vision (94%) and pain (79%) were the most common symptoms. Most were pseudophakic (79%), including posterior chamber IOLs (77%) and anterior chamber IOLs 2%. Wound complications were present in 23 (68%) of cases, included fibrinous reaction (56%), vitreous wick (29%), and wound leak (12%). An afferent pupillary defect was present in 21% and a hypopyon in 53% with a mean size of 0.57±0.7mm. The initial BCVA ranged from 20/200 to light perception and was·e5/200 in 12%, between 5/200 and hand motion in 35% and·d hand motion in 53% of all patients.

**Table 1:** Clinical Characteristics of Studied Cases at Presentation.#

Variable	Data
Age, year	
Mean±SD	74.3±15.24
Range, year	3-92
	
Sex	
Male	18 (53)
Female	16 (47)

Eye	
Right	16 (47)
Left	18 (52)

Interval from Previous Surgery, Month
Mean±SD	51.5±58
Range, month	0.03-249.3

Pain	27 (79)

Decrease Vision	32 (94)

Duration of Symptoms, Day
Mean±SD	2.88±4.93
Range, day	1-30

Afferent Papillary Defect	7 (21)

Hypopyon	18 (53)
Size if present, mm Mean±SD	0.57±0.67

IOL* Status
None	7 (21)
Anterior Chamber	1 (3)
Posterior Chamber	26 (76)

Wound Complication
Fibrinous reaction	19 (56)
Vitreous presentation	10 (29)
Leakage	4 (12)

Visual Acuity Range
>5/200	4 (12)
5/200 to HM**	12 (35)
HM	18 (53)

# unless otherwise indicated, data are represented as number (%) of patients;

* intraocular lens;

** hand motion

### Culture Results

All patients had a positive vitreous culture by inclusion criteria (Table 2).

**Table 2:** Distribution of Isolated Organisms According to the Source of Specimen.

Cultured Organisms №(%)						
Source of Specimen	Gram-positive, coagulase-negative staph	Gram-positive, coagulase-positive staph	Gram-positive, Streptococcal species	Gram-positive bacilli	Gram-negative species	Total

Vitreous	8 (20%)	2 (5%)	22 (55%)	2 (5%)	6 (15%)	40 (100%)

Aqueous	3 (33%)	0	5 (56%)	1 (11%)	0	9 (100%)

An anterior chamber culture was obtained in 44% of cases; 67% were positive. Organisms from the anterior chamber differed from that from the vitreous in 20% of cases. Streptococcus species were the most common vitreous isolates (55%), Str. viridans and faecalis were the most frequently isolated strains, 18.2% each.

Gram-positive, coagulase negative staphylococcus (most commonly S. epidermidis) was the second most commonly isolated vitreous microorganisms (20%). Organisms recovered from A/C cultures had a similar frequency of distribution (table 2). Of all anterior chamber and vitreous cultures, polymicrobial growth was noted in 9 of 34 cases (27%): two organisms were isolated in eight of them and three organisms were isolated in one case. Organisms recovered from the specimens were sensitive to the administered antibiotics in 32 (94%) cases.

Initially, 12 (35%) cases underwent tap-injection with vitrectomy while the remaining 22(65%) had tap-injection alone. Later PPV was done for 11(32%) more cases, 8 of them were from the tap-injection group. Overall PPV was done in 20(59%) cases. All three cases that underwent reinjection were from the tap-injection group.

Eighteen patients (53%) were treated as inpatients with a mean stay 1.56 ± 1.9 days.

Antimicrobials and corticosteroid usage (Table 3).

**Table 3:** Frequency of Agents Used in the Treatment of Studied Cases.

	Drug Delivery Method %			
Agent	Intravitreal injection	Subconjunctival injection	Topical drops	Systemic

Vancomycin	100	50	91	20.6

Ceftazidime	94	35	53	12

Gentamycin	3	21	3	6

Tobramycin	3	0	21	0

Corticosterold	56	50	59	0

Ciprofloxacin	0	0	0	15

Maxitrol	0	0	3	0

Intravitreal agents used included vancomycin (100%), ceftazidime (94%) and dexamethason (56%). Subcojunctival injections were used in 21 (62%) cases and included vancomycin, ceftazidime and dexamethason (50%, 35%, and 50% respectively). Topical agents included vancomycin, ceftazidime and prednisolone acetate. Oral or intravenous antibiotics were used in 14%. Systemic corticosteroids were not administered to any patient.

### Visual Results

The final BCVA was 5/200 or better in 50% compared to 12% at initial examination (p=0.001).A final BCVA of·e 20/400 resulted in 16 (47%) of 34 patients. There was no light perception (NLP) in 8 (24%) patients. The 14 cases managed without PPV included 7 (50%) had visual acuity of·e20/400; 4 (33%) of the 12 eyes managed with a PPV had BCVA of ≥20/400 (p= 0.45). (Table 4). Final BCVA of·e20/400 resulted in 5 (83%) gram-positive, coagulase-negative staphylococcal species cases, and 7 (47%) streptococcal species cases (P=0.18).

**Table 4:** Summary of Patients Data at the End of Study Observation.

Variable	Data
Follow Up Interval, Month	
Median	15.29 ± 18.28
Range	0.9-78.1

Final Visual Acuity, No(%)	
20/50	5 (15)
<20/50 to 5/200	12 (35)
<5/200 to HM*	5 (15)
<HM	12 (35)

Reinjection, No(%)	3 (9)

Later PPV**, No(%)	11 (32)

Patients Admitted, No(%)	18 (53)

Inpatient Stay, Day	
mean±sd	1.56 ± 1.93

* hand motion;

** pars plana vitrectomy.

Inadequate response to therapy necessitated further surgery of evisceration or enucleation in 7 (21%) cases. Culture results of those cases were Gram negative organisms in three cases (H.influenzae, Serattia marcescens and pseudomonas aeruginosa), gram positive organisms (streptococcus salivaris, sanguinis and beta-hemolytic) three cases, and both Str. mitis with S. epidermidis in one case.

At final follow up one case had dislocated intraocular lens with retinal detachment, two cases had epiretinal membranes and one case left with a phthitic eye.

## DISCUSSION

The results of this study are consistent with other reports finding BRE as the second most common form of infectious endophthalmitis, after acute and chronic post-cataract surgery Endophthalmitis.^1^ BRE usually develop late (>1month) but in our series 15% of the cases had BRE earlier (< 2weeks). Possible reasons for an earlier onset might be a high frequency of polymicrobial growth of virulent bacteria. In addition, pseudophakia, vitreous wick, and bleb leakage have been reported as potential risk factors that may contribute in the development of BRE^10^,^15^,^16^ and these factors were present in our cases (79%, 29%, and 12% respectively). As reported in cases of endophthalmitis following cataract surgery^17^; hypopyon present in a surprisingly low proportion (53%) and, therefore, the significance of its absence must not be overemphasized.

The study results are also consistent with previous reports that streptococcus species (55%) and coagulase negative staphylococci (20%) are the most common infecting organisms in BRE.^18^,^19^ Our results coincide with the findings in EVS that vitreous is a richer source of positive cultures than the aqueous^20^. The frequency of polymicrobial growth (27%) was higher in the current study than that reported by the EVS for post-cataract surgery endophthalmitis cases (9.3%)^21^ and may have played a role in the visual end results were NLP vision was higher (24%) in BRE cases compared to EVS (5%). A high rate of NLP vision (18%) after post-operative endophthalmitis was reported by others.^22^

Successful management of infectious bacterial endophthalmitis depends on timely diagnosis and prompt institution of appropriate therapy.^23^ No single antibiotic provided coverage for all of the microbes isolated from eyes with endophthalmitis,^24^ but cultured microorganisms were sensitive to the antibiotics used in 94% of cases in the current study. Accordingly, empiric, broad spectrum intravitreal antibiotic therapy appears to be justified without awaiting culture results. Intravenous antibiotics were uncommonly used and most were managed in outpatient setting; when admitted, the stay was brief (1.6 ± 1.9 days).

The initial treatment regimen most commonly used was vitreous tap-injection without PPV (65%). Later PPV was performed in 8 (36%) cases; meaning 59% of the studied cases underwent PPV. Busbee BG et al reported that BRE cases treated with tap-injection had significantly worse final visual acuity and higher rate of NLP vision than patients treated with tap-PPV-injection,^25^ but in the current report the difference in final BCVA of the two groups was not statistically significant. The visual outcome of endophthalmitis cases occurring after cataract surgery has been reported to be worse with growth of streptococcal species than with growth of staphylococcal ones,^26^ but the current study of BRE did not find a significant difference (p=0.18).

Complications are common with BRE, as 21% of cases underwent evisceration or enucleation secondary to phthisis, pain and/or poor vision (LP to NLP), and only 47% of the cases had visual acuity of 20/400 or better. The observed frequent polymicrobial growth of more virulent infecting organisms and recorded potential risk factors (pseudophakia, vitreous wick and bleb leak) may contribute in BRE clinical progression and severity that result's in poor visual outcome.

## CONCLUSION

Despite prompt and intensive treatment of patients with BRE the end result remains disappointing. In BRE Streptococcal species are the most common causative organism. Based on culture and antibiotic susceptibility results, initial treatment of BRE with intravitreal vancomycin and broad spectrum antibiotic like ceftazidime appears to be justified until culture results are obtained. Neither tap-injection with PPV nor tap-injection without PPV proved superior in the management of this condition.
